# Initial Resuscitation of a Multisystem Trauma Patient Following a Fall From Height: A Complete Simulation Scenario for Medical Students

**DOI:** 10.7759/cureus.13013

**Published:** 2021-01-30

**Authors:** Leah O Grcevich, Maxwell J Jabaay, Benjamin T Leicht, James Lyons

**Affiliations:** 1 Department of Research, Alabama College of Osteopathic Medicine, Dothan, USA; 2 Department of Clinical Sciences, Alabama College of Osteopathic Medicine, Dothan, USA

**Keywords:** simulation in medical education, simulation design, emergency medicine resuscitation, trauma management, traumatic injury, pneumothorax (ptx), femur and fracture, life-threatening bleeding, medical education

## Abstract

Management of a complex trauma patient is a critical skill for medical students, particularly during a general surgery or emergency medicine clerkship. However, gaining proficiency with this skillset may be challenging without prior medical or simulation experience. The aim of this technical report is to present a comprehensive high-fidelity medical simulation of a polytraumatized patient with numerous injuries sustained from a 20-foot fall. As the scenario unfolds, students identify multisystem injuries including acute hemorrhage, femur fracture, tension pneumothorax, and traumatic brain injury. The case was designed as an assessment tool to evaluate the knowledge of preclinical medical students obtained through a one-day workshop on the primary survey. This technical report provides simulation designers with a premade script, flowchart, labs, images, and supplies needed to successfully recreate the case.

## Introduction

Traumatic injury represents a significant public health concern in the United States, with 20 million trauma-related discharges annually, representing 4.4% of all-cause hospital discharges between 2000 and 2011 [1]. Traumatic injury is the number one cause of shortened lifespan in those under the age of 65 years [2]. The most common mechanism of trauma is a fall (comprising 47% of all injuries) and is common among all ages [1,3]. In recent years, other mechanisms of injury such as motor vehicle collisions have seen a decrease in case fatality, while mortality from falls has increased by 46% [4]. Trauma patients have worse long-term survival than age-matched controls, and long-term survival is correlated with existing comorbidities and location of discharge (home versus skilled nursing facility) [5]. Multisystem trauma patients represent some of the most complex cases received by trauma physicians. Within the context of this paper, multisystem trauma is referring to a mechanism of injury involving two or more body systems or regions [6].

The following simulation scenario involves the management of a multisystem trauma patient whose injuries were incurred following a fall from 20 feet. This scenario was originally developed as an assessment tool for a comprehensive one-day curriculum, in which medical students were instructed on how to perform the primary survey [7]. Goals for the event were based on the five components of the primary survey including evaluation and management of airway, breathing, circulation, disability, and exposure (ABCDE). This case functioned as a final assessment tool; it was designed to test students’ ability to think critically and apply key components of the primary survey to treat a polytraumatized patient.

## Technical report

Learning objectives

Each learning objective corresponds with a component of the primary survey (ABCDE):

1. A - Identify a patent airway.

2. B - Identify a tension pneumothorax based on auscultatory and chest x-ray findings.

3. B - Verbalize or perform a needle decompression to treat a tension pneumothorax.

4. C - Identify hypovolemic shock and its proximal cause.

5. C - Treat the underlying cause of hypovolemia by applying a tourniquet or pressure dressing, and support systemic circulation by administering fluids or blood products.

6. D - Identify a traumatic brain injury.

7. E - Remove the patient’s clothing to identify injuries.

8. Perform a concise handoff. 

Context

This simulation was run as part of a workshop on the primary survey [7]. The intended audience was preclinical medical students but could be adapted to include higher level providers with some additions to the case. This case was run in a medical school simulation center as if the patient was just transferred to an emergency department (ED) trauma bay from emergency medical services (EMS). This case could be adapted for in-situ simulation in a variety of inpatient and outpatient environments.

The case is designed so that each component of the primary survey needed to be addressed based on the patient’s evolving condition. The scenario is presented as a progressively unfolding encounter so that after a successful intervention is made the team must reassess ABCD as the patient continues to decompensate. Students approach this simulation in teams of five. Within each group, every student has a predefined role: team leader, history taker, physical examiner, proceduralist, and scribe. In addition, each simulation room includes one healthcare actor (HA) taking on the role of an ED nurse. The role of the HA is to provide guidance if the team veers too far off track and allow learners to progress through the scenario before the patient deteriorates [8]. 

Inputs

The following is a list of props, personnel, and equipment required to run the simulation. Alternatives and substitutions are recommended where applicable.

**Figure 1:**
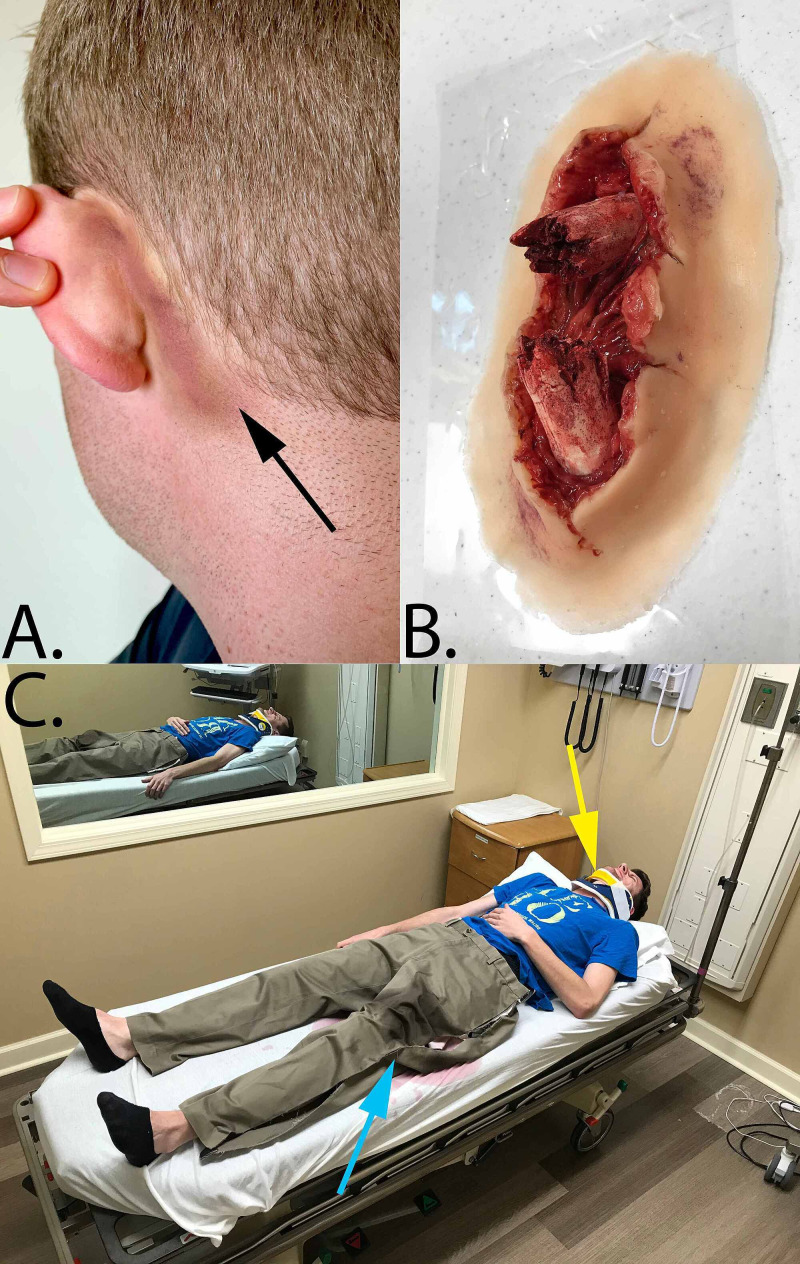
Simulation props (A) The black arrow highlights dark makeup which was applied behind the ears to mimic retroauricular ecchymosis. (B) Silicone prosthetic was applied to the thigh of the SP to simulate a compound femur fracture. (C) At the beginning of the scenario, students encounter a human SP (or high-fidelity simulator) fully dressed in a hospital bed with cervical collar in place. The yellow arrow is pointing to the location of the cervical collar in the image. The light blue arrow highlights the pre-cut clothing to facilitate easy exposure of the patient. SP, Standardized patient.

1. A standardized patient (SP) who had memorized a patient script (Appendix A) for the case and who was trained in the overall case flow (Appendix B) in order to cue the participants if needed

2. An ED nurse if available or a HA trained on the case flow embedded in the scenario to control the situation, should a team become preoccupied with non-vital information

3. Simulation facilitator to provide vital signs, imaging, etc. to the monitor in the room or provide paper copies (labs included in Appendix C and imaging included as figures throughout)

4. Purple and black makeup applied behind the ears to indicate battle signs (Figure 1A)

5. Prop open femur fracture (Figure 1B) with an attached prop blood bag filled with fake blood (see Appendix D)

6. Prop clothing that can be cut with trauma shears or pre-cut (Figure 1C)

7. Cervical collar positioned on the patient (Figure 1C)

8. Training tourniquet or gauze

9. Red and purple makeup applied to right chest overlying site of tension pneumothorax

Preparation timeline

1. Two weeks prior to beginning this simulation, SPs are provided their script and brought back for a training session to explain the flow of the case and to practice reacting to the participants’ questions.

2. Prior to the beginning of the case, each member of a team of five chooses to take on one of the following roles: team leader, physical examiner, history taker, scribe, and proceduralist.

3. The case begins with the team receiving a one-time oral report of the patient from the EMS. After obtaining the report, participants report to the simulation center or emergency room (ER) trauma bay.

4. Encounter is initiated by the team captain’s introduction to the patient and continues with other team members completing a history and assessment.

5. The team assesses and treats the patient’s life-threatening injuries. Upon reassessment, a new trauma sequela is presented, and the team must act accordingly. The patient will begin to deteriorate, or there will be a change in the patient’s status only after the desired task is accomplished.

6. At the completion of the case, students report to be debriefed.

Simulation scenario

The case begins outside of an emergency room resuscitation bay, with the students receiving the following prerecorded EMS radio report (Video 1):

EMS report: “This is Houston county rescue five coming to your facility with a priority one trauma alert. We have a 25-year-old male {Glasgow Coma Scale score} GCS 13, alert and oriented two out of three, negative to place, who fell from a height of 20 feet, and his left leg has taken most of the impact. The patient has a compound femur fracture. The patient has lost close to one liter of blood, and bleeding is not controlled. The patient's vitals are heart rate 130, blood pressure 106/88, respiratory rate 22, and SpO2 is 97% on room air. IV has been placed, and we have taken C-Collar precautions. We are three minutes away.” 

**Video 1:** EMS radio report EMS, Emergency medical services.

Students are then allowed to enter the patient room. Vital signs, patient actions, expected student responses, and operator notes for the initial stage are included in Table 1. 

**Table 1:** Initial hypotensive state Student actions include a list of actions that must be completed by the students in order to move on to the next phase of the simulation. If students struggle to complete the requisite actions, these actions may be prompted by the standardized patient (by repeating the quotation listed within the operator notes) or by the healthcare actor.

Vital signs	Patient status	Student actions	Operator notes
Pulse 138, regular	The patient initially has eyes closed but will be able to open eyes and answer questions when asked	Obtain a focused history	If students do not know what to do, the patient may state the following: “please help me - I think I am going to bleed out.”
Blood pressure (BP) 104/72	Bleeding wound	Cut off the patient’s clothing	If student’s fail to address bleeding wound, the patient states the following: “please, I’m going to die if you don’t stop the bleeding.”
Respiratory rate (RR) 22	Patient is aware of events preceding injury.	Complete a rapid head-to-toe physical	If radiographic imaging of the head is ordered, hold until the final state with altered mental status.
Pulse oxygen (SpO2) 94%	Pain is 8/10.	Address bleeding (either pack or apply tourniquet) and administer fluids	If lower extremity x-ray is ordered, it may be provided at this time.

As students enter the room, they find a distressed patient with uncontrolled bleeding associated with an open fracture, indicating life-threatening hemorrhage. This is accomplished by using a prop open femur fracture (Figure 1B) connected to a fake blood bag (Appendix D). The flow of blood from the wound is controlled by the SP pressing their back on the blood bag.

**Figure 2:**
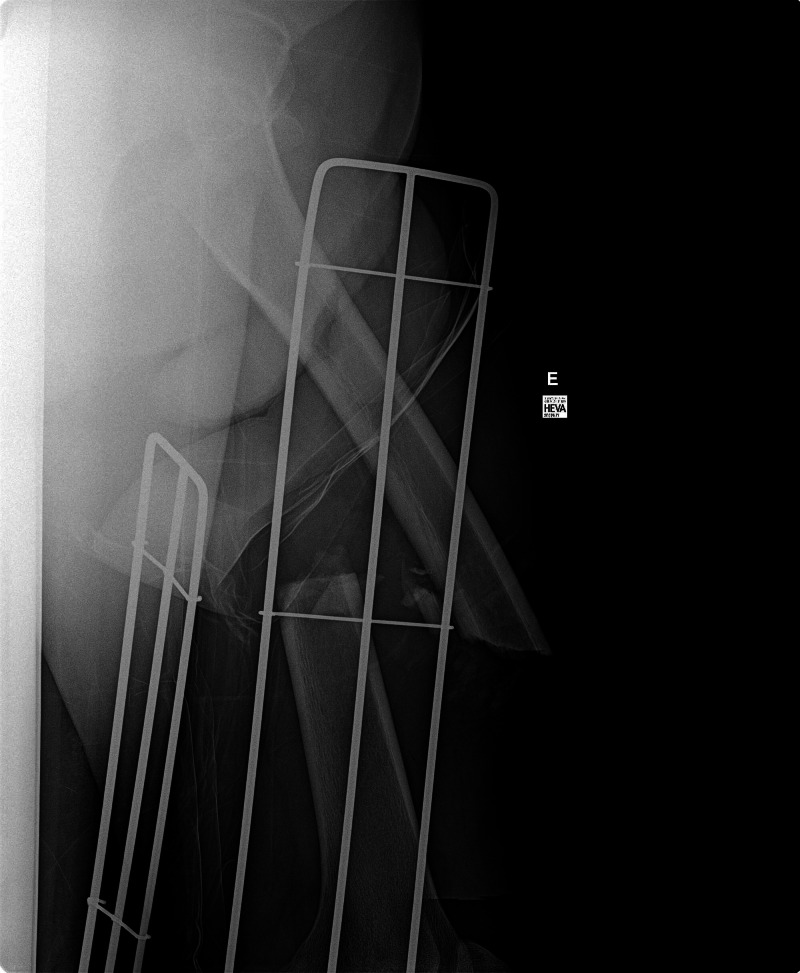
X-ray showing open displaced femur fracture Case courtesy of Prof. Cláudio Souza, Radiopaedia.org, rID: 13772.

The focus of this stage is for students to identify massive hemorrhage and begin the primary survey with circulation rather than airway management. Patients in need of resuscitation usually follow an algorithm of airway, breathing, circulation (ABC); however, in cases of massive hemorrhage, it is a standard practice to prioritize circulation before airway and breathing (CAB) as oxygenation and ventilation attempts would be ineffective without adequate blood volume [9]. Actions required to move on to the next stage may be modified based on the level of the students. First- and second-year medical students are expected to identify and address the patient’s bleeding through the application of a pressure dressing or tourniquet. These actions must be performed in conjunction with supporting the patient’s blood pressure through the administration of fluids or blood products. Initial stabilization of the patient should take precedence prior to obtaining imaging. If an x-ray was ordered following completion of the primary survey and appropriate interventions, imaging would confirm the diagnosis of open displaced femur fracture (Figure 2).

Following the primary assessment and hemorrhage control, the patient becomes confused and short of breath, as outlined in Table 2. 

**Table 2:** Intermediate hypoxic state The table includes patient vitals and corresponding actions of all participants during the intermediate hypoxic state. HR, heart rate; BP, blood pressure; RR, respiratory rate; SpO2, pulse oxygen.

Vital signs	Patient status	Student actions	Operator notes
HR 118	Patient is becoming more confused.	Auscultate lungs, and identify absent breath sounds on right	If students do not apply oxygen or order a chest x-ray after 1-2 minutes, the patient may state the following: “it feels like I have one lung.”
BP 114/80	Patient complains of shortness of breath and chest pain	Administer supplemental oxygen	If students do not order chest x-ray after another minute, healthcare actor asks “did we order all of the necessary trauma films?”
RR 24		Order chest x-ray	
SpO2 90%			
Vital signs change after the chest x-ray is ordered			
HR 142	Patient is more confused (alert and oriented to person, place, and time but not preceding events).	Recognize pneumothorax on x-ray	Provide students with a chest x-ray image (digital or paper copy).
BP 110/80	Patient appears physically agitated and has increased work of breathing.	Needle decompression	If students do not know what to do after several minutes, radiology will call stating the following: “This is radiology. We wanted to inform you that on the STAT chest x-ray we see a tension pneumothorax and advise that you perform a needle decompression.”
RR 26			
SpO2 85%			
Following needle decompression SpO2 95%			

**Figure 3:**
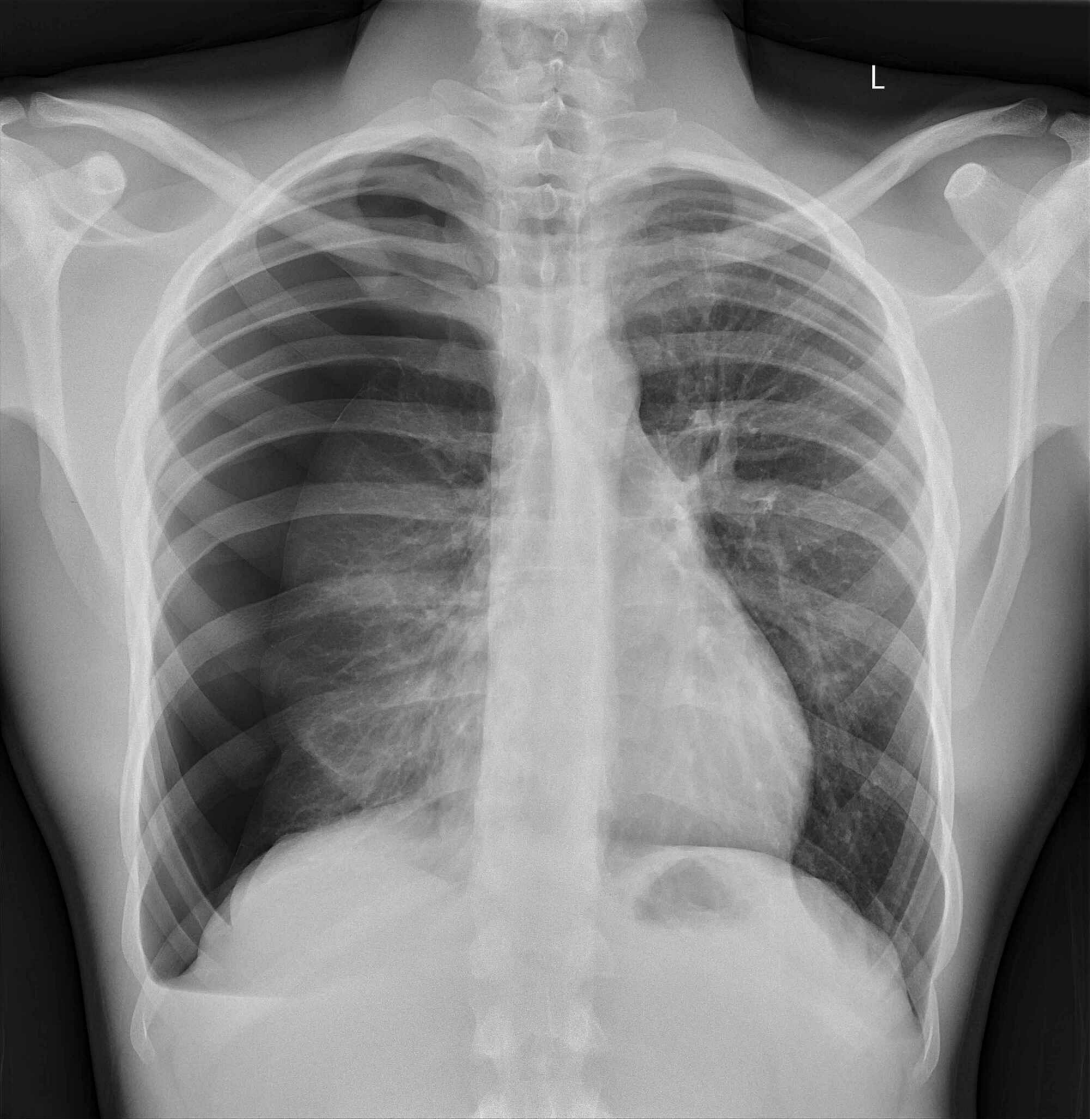
Chest x-ray showing tension pneumothorax Case courtesy of Dr. Ana Brusic, Radiopaedia.org, rID: 60551.

Decompensation is a signal for students to reassess ABCD, to identify what is contributing to a change in the patient’s condition. Students may attempt to treat hypoxemia by applying supplemental oxygen. This action does not completely resolve the patient’s symptoms or oxygen saturation (SpO2), in order to prompt further examination by the student. Upon examination of the airway and auscultation of lungs, students are expected to identify a tension pneumothorax as the cause of the patient’s shortness of breath and chest pain. Chest x-ray is not required to confirm the diagnosis, but a representative image is included if requested during the scenario (Figure 3).

Physical exam and vital signs concordant with tension pneumothorax in this scenario include decreased ventilation, hyperresonance to percussion, contralateral tracheal deviation, hypoxia, tachypnea, tachycardia, and rarely hypotension as a late complication [10]. Unstable patients may require emergency treatment by needle thoracostomy prior to radiographic confirmation of the diagnosis [11]. Verbalization of needle or chest tube thoracostomy is appropriate for this level of student. Advanced learners may demonstrate either skill on a task trainer.

After the verbalization of thoracostomy, the patient appears drowsy and begins complaining of head pain (Table 3).

**Table 3:** Altered mental status-secondary to traumatic brain injury (TBI) CT, Computed tomography; HR, heart rate; BP, blood pressure; RR, respiratory rate; SpO2, pulse oxygen.

Vital signs	Patient status	Student actions	Operator notes
HR 110	Patient is complaining of head pain.	Order head CT	If students are unsure of next steps, healthcare actor may state “do you think we need additional help” or “I think we need to call neurosurgery.”
BP 110/80	Patient is confused (now alert to person only).	Identify epidural bleed on imaging	
RR 20		Consult neurosurgery, giving a brief report on the patient’s condition	
SpO2 95%			

**Figure 4:**
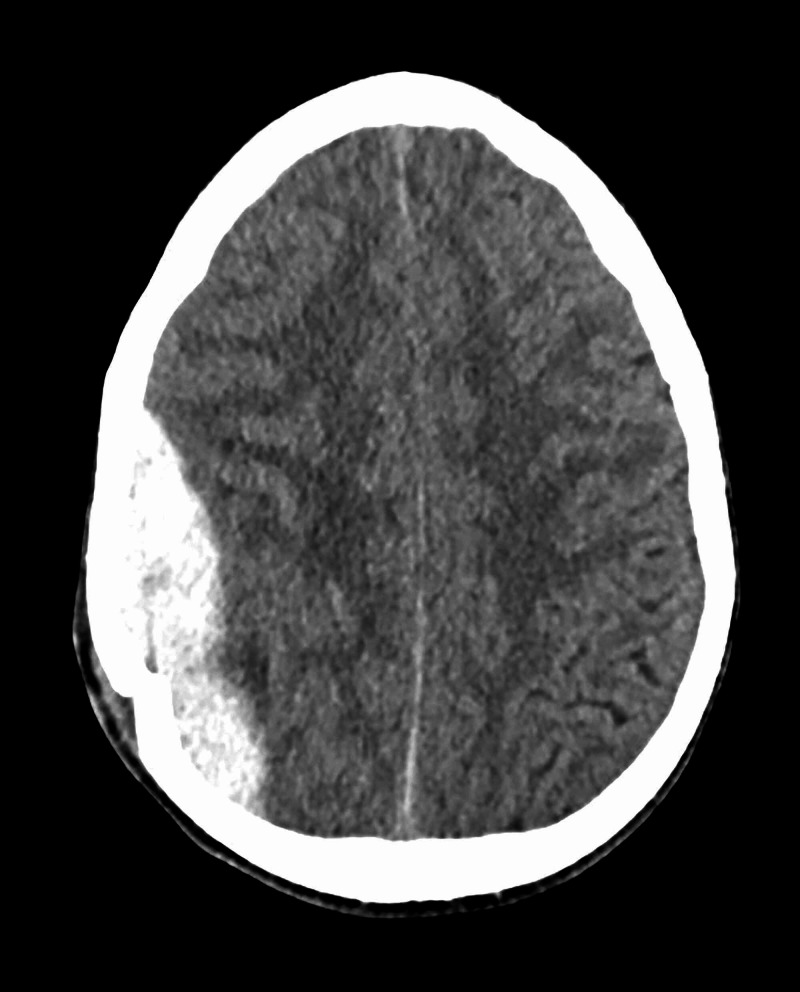
Epidural hematoma underlying a depressed skull fracture Case courtesy of Dr. Michael P Hartung, Radiopaedia.org, rID: 74815.

Prompted by a change in neurologic status, students may assess impairment of consciousness using the Glasgow Coma Scale (GCS). The GCS is a clinical tool widely used in the treatment of trauma patients to simply communicate the severity of a patient’s condition [12]. The patients opens their eyes spontaneously, has confused speech but follows commands, which equates to a GCS of 14. Students may follow up the GCS by assessing if the patient is alert and oriented to person, place, time, and preceding events (A/Ox4/4). As students reassess the patient's level of orientation, the patient is alert and oriented (A/O)x1/4 (previously A/Ox3/4), negative to time, place, and preceding events. Over time the patient becomes more somnolent and difficult to arouse. Physical examination reveals retroauricular and mastoid ecchymosis, colloquially termed “battle signs.” Although battle signs usually present late in the clinical course, this physical exam finding is included for a simulation with novices in order to facilitate an association between this finding and significant neurologic injury. Additionally, this finding emphasizes the importance of a thorough secondary survey after completing the primary "ABCDE" assessment. A decrease in the level of consciousness in addition to head pain in the setting of trauma should prompt learners to consider a traumatic brain injury (TBI) and order computed tomography (CT) of the head (Figure 4). The goal of this state is for students to identify an epidural hematoma and consult neurosurgery.

Debriefing

Debriefing of this simulation highlights elements of the assessment and management of a multisystem patient relevant at the preclinical medical student level. This includes identification and acute intervention of hemorrhage control from a femur fracture and thoracostomy for a tension pneumothorax while also identifying a TBI. Designing a multisystem trauma scenario assists in bolstering foundational anatomical knowledge and assessment of medical students’ abilities to think critically in an emergent situation with an unstable patient.

Compound fractures are most often seen in middle-aged males with crush injuries due to motor vehicle accidents and falls from standing heights [13]. In patients with compound fractures, reduction in the ED may be required to achieve hemostasis [13]. Once the patient is stabilized they should receive prophylactic antibiotics and updated tetanus immunization as indicated. Definitive intervention for the fracture would include wound irrigation and debridement followed by surgical intervention for fixation and wound closure [13].

In addition to an obvious femur fracture, this simulation includes a tension pneumothorax resulting from a rib fracture. This is included to encourage students to examine a patient closely for injuries after sustaining a trauma significant enough to fracture one of the strongest bones in the body. In addition, students are able to identify compromised breathing and determine an appropriate intervention. The diagnosis of tension pneumothorax may be made clinically or confirmed with imaging. Appropriate management includes aspiration with needle or chest drain followed by careful monitoring [14].

Lastly, it is critical that medical students suspect traumatic brain injuries in trauma patients with altered mental status since TBIs are the leading cause of death in this population [15]. In this scenario, students were provided with physical examination findings and imaging indicative of intracranial mass effect secondary to a basilar skull fracture and an ensuing epidural hematoma. It is well documented that mass effect has short- and long-term cognitive complications, such as changes in mental status, mood, and diminished health-related quality of life [16]. The clinical features of basilar skull fractures may present acutely in nonspecific ways, including altered mental status, nausea, vomiting, and cranial nerve deficits [17]. Other findings, such as hemotympanum, cerebrospinal fluid rhinorrhea or otorrhea, periorbital ecchymosis, and retroauricular ecchymosis, may not present until hours or days following the injury [17].

## Discussion

This simulation was designed not only to test the students’ medical knowledge but also to provide exposure to the continuum of care required to resuscitate a trauma patient. In order to mirror the environment (ED) in which the skill being taught (primary survey) would be practiced, careful consideration was given to the team-based approach for trauma patients; this includes pre-hospital EMS, ED trauma teams (including physicians, nurses, and technicians) as well as definitive care providers such as surgical and intensive care teams. Specifically, EMS crews have critical information about the mechanism of injury, timing of events, other patients at the scene, and tools that were required for extrication [18]. The role of EMS was given consideration in this scenario by including a handoff from the pre-hospital crew to the ED team via radio report.

Following receipt of a radio report, students were allowed to enter the room and were told to evaluate and treat the patient’s life-threatening injuries. The expected actions for students to take were based on the primary survey consisting of assessment and management of ABCDE. Interventions were based on specific injuries identified through the primary survey, the most significant of which were traumatic brain injury, tension pneumothorax, and compound femur fracture. These conditions were progressively revealed to the students in order to emphasize the importance of reassessment as well as mitigation of cognitive overload.

Given the experience of targeted learners, trained HAs playing the role of ED nurses were included in the encounter. These individuals were trained on the flow of the case and instructed on when to intervene. Their interactions allowed the case to unfold in the order in which it was designed and provided the students some insight on next steps without taking away from the learning experience. Additionally, they limit distractions and nonproductive discussion. For more experienced learners, this scenario could be augmented by replacing trained HAs with clinical support staff not pre-briefed on the case flow. This would maintain the realism of the scenario and add a degree of difficulty.

## Conclusions

Fall injuries are a common occurrence in adults of all ages. These events can lead to a variety of injuries impacting most body systems. As such, these scenarios are a valuable learning tool for medical students, residents, and seasoned physicians alike. This technical report provides a turn-key medical simulation that can be modified for a broad audience of students. Through methodical application of the primary survey, students can learn to effectively diagnose, manage, and treat a complex scenario in a safe learning environment.

## Figures and Tables

**Figure 5:**
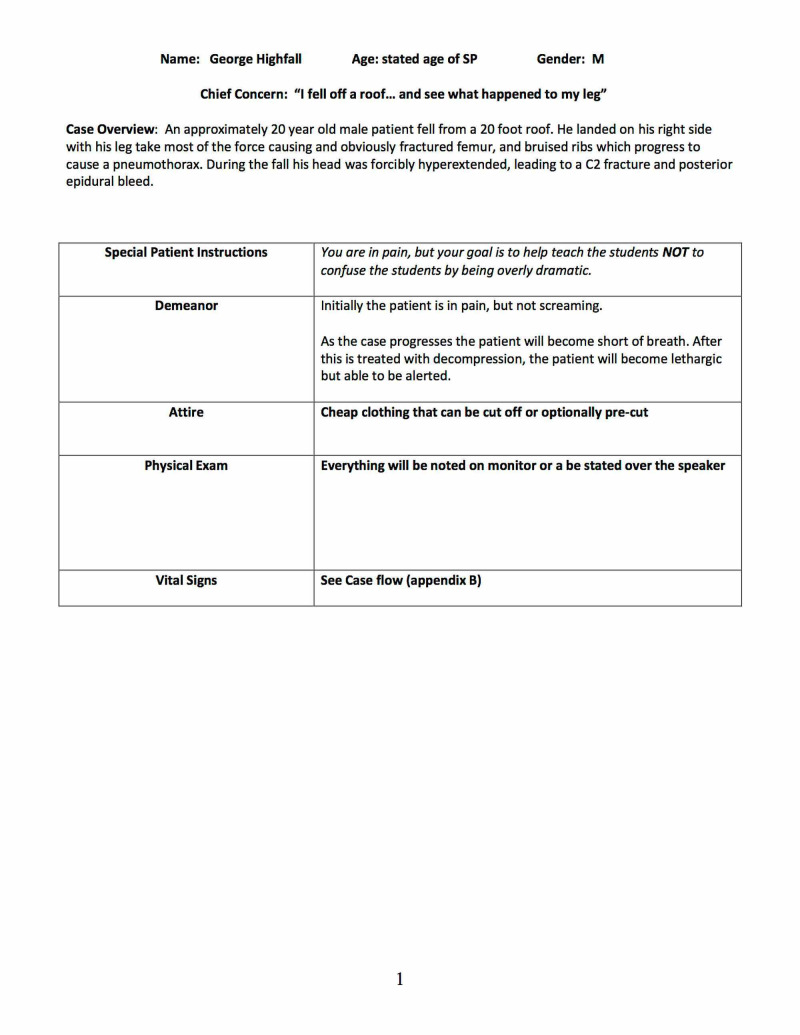
Script Page 1

**Figure 6:**
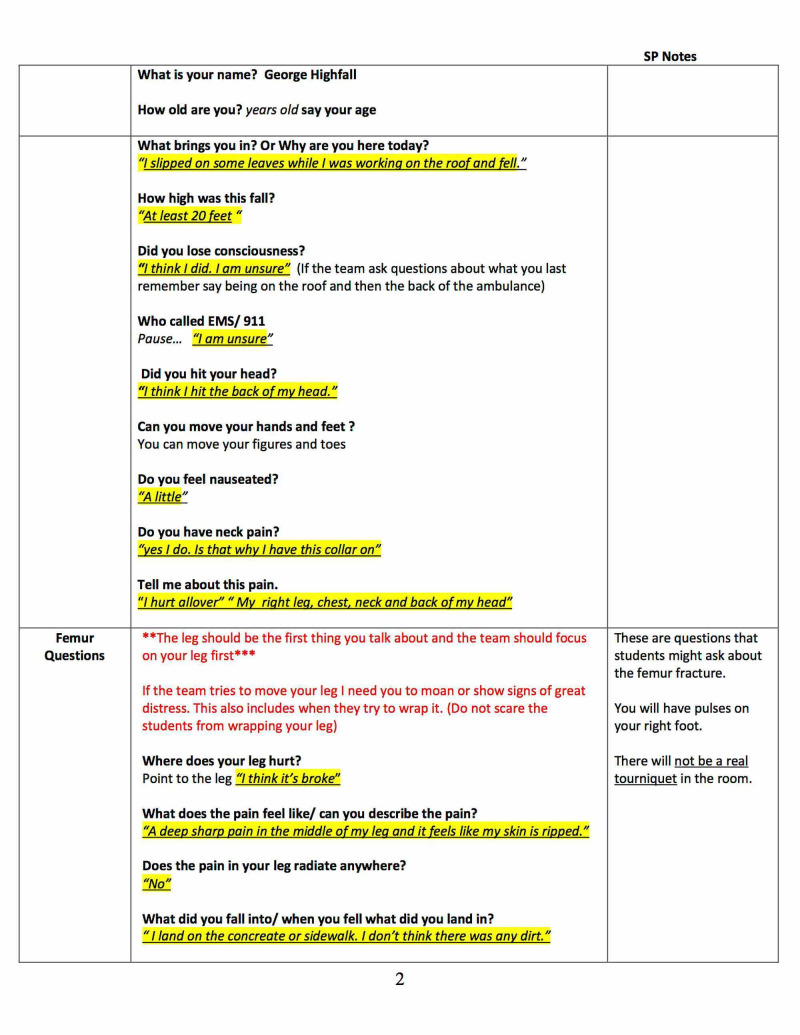
Script Page 2

**Figure 7:**
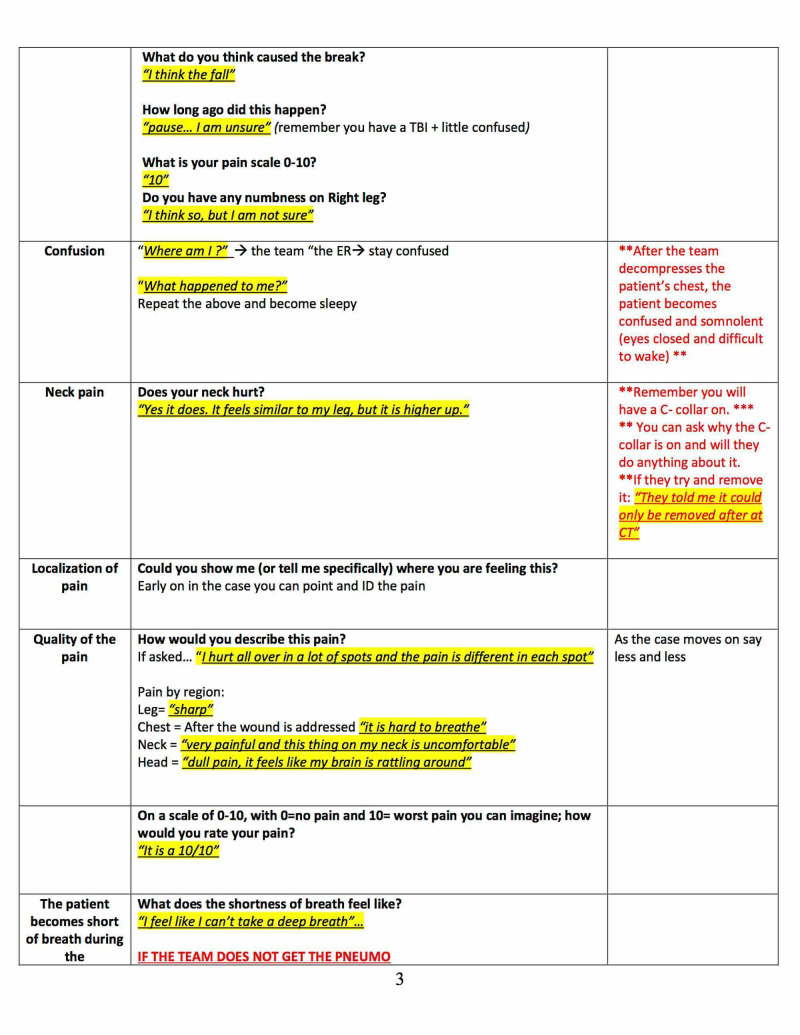
Script Page 3

**Figure 8:**
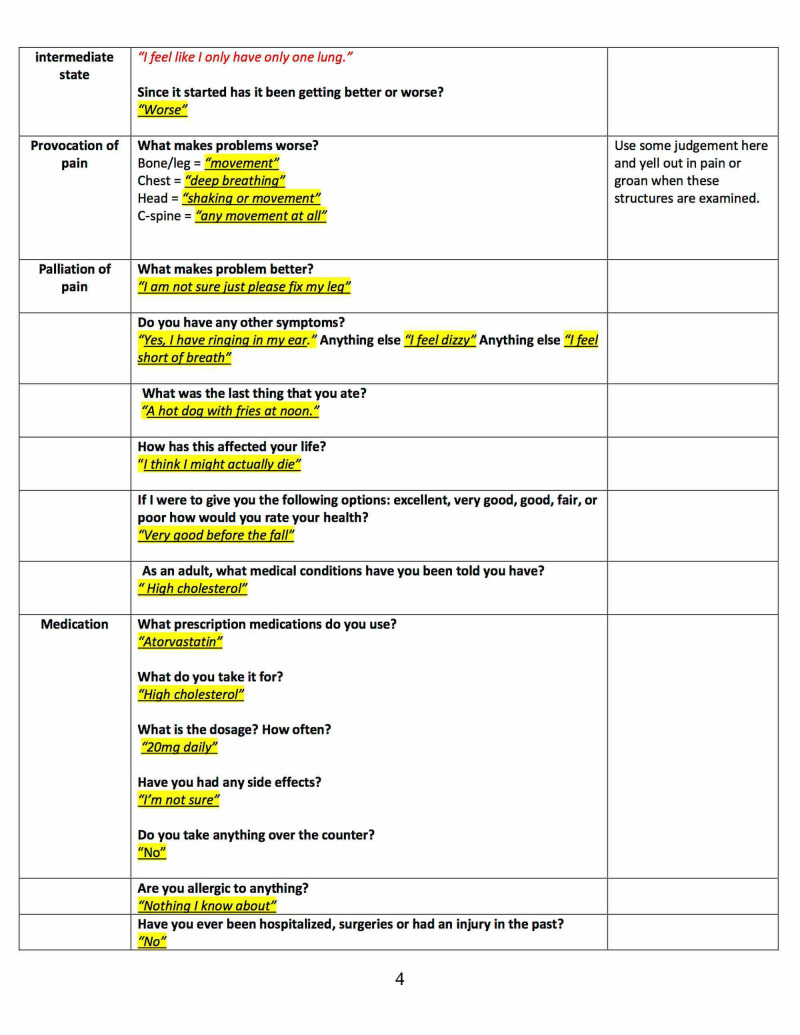
Script Page 4

**Figure 9:**
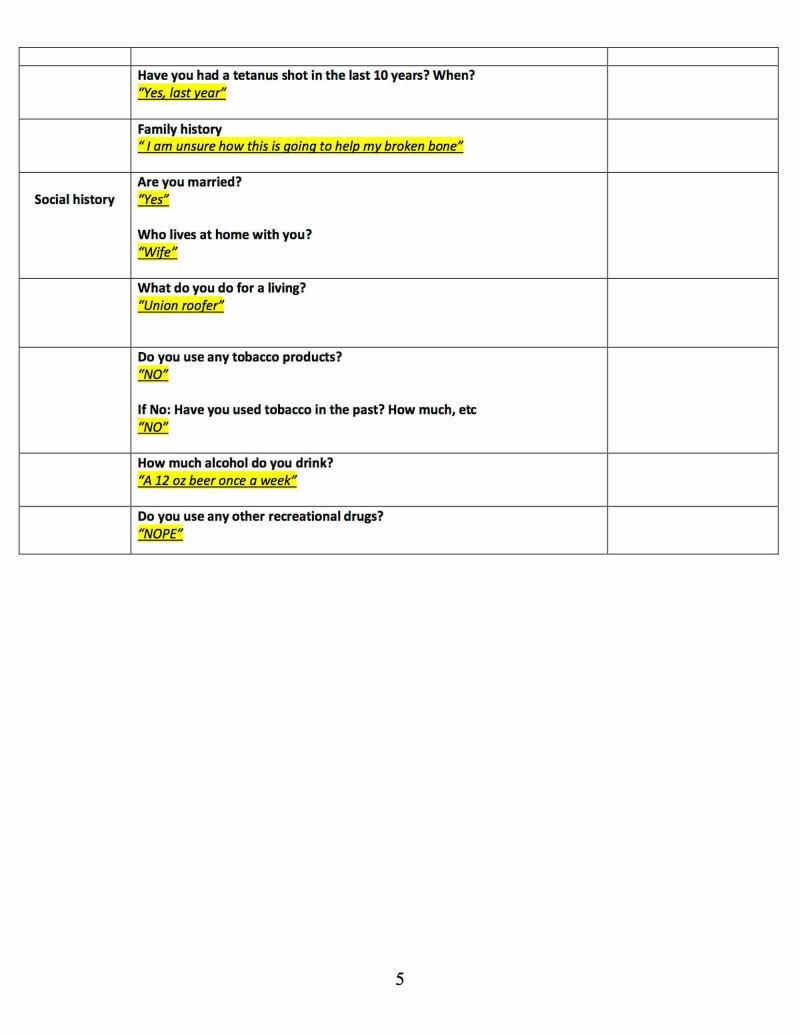
Script Page 5

**Figure 10:**
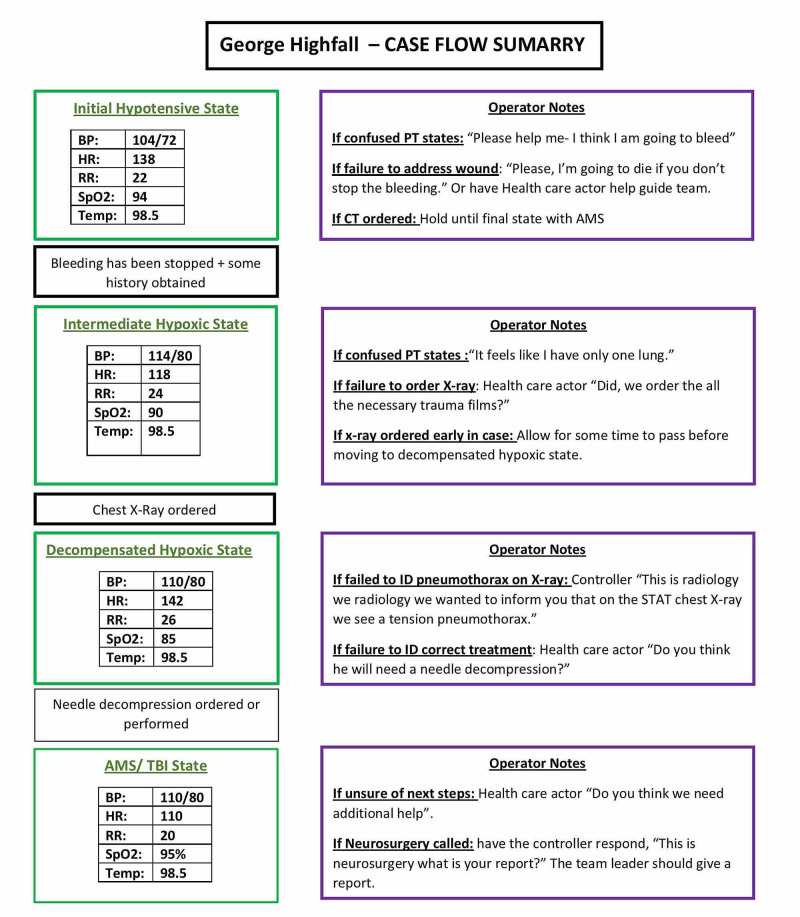
Simulation case flow

**Figure 11:**
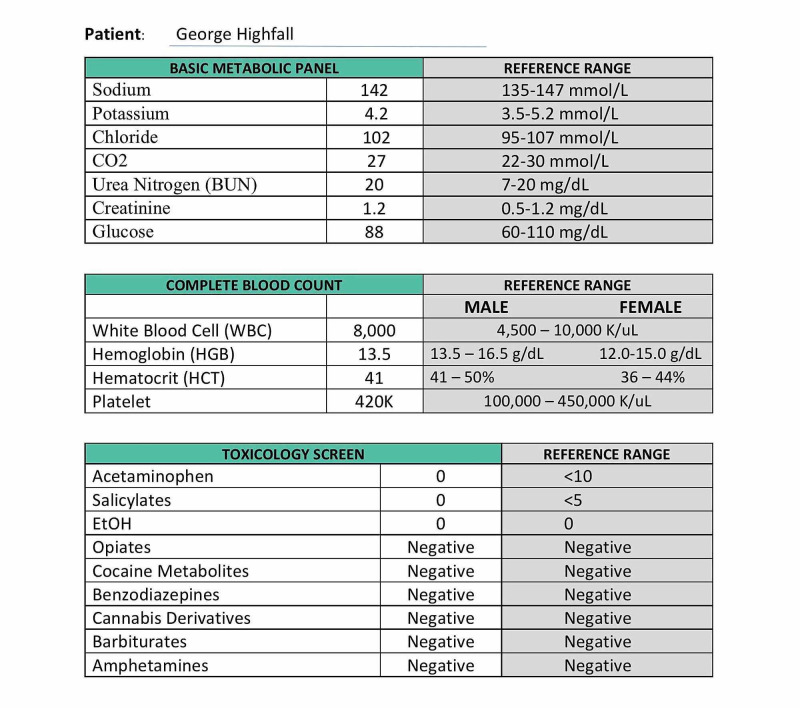
Laboratory results

**Figure 12:**
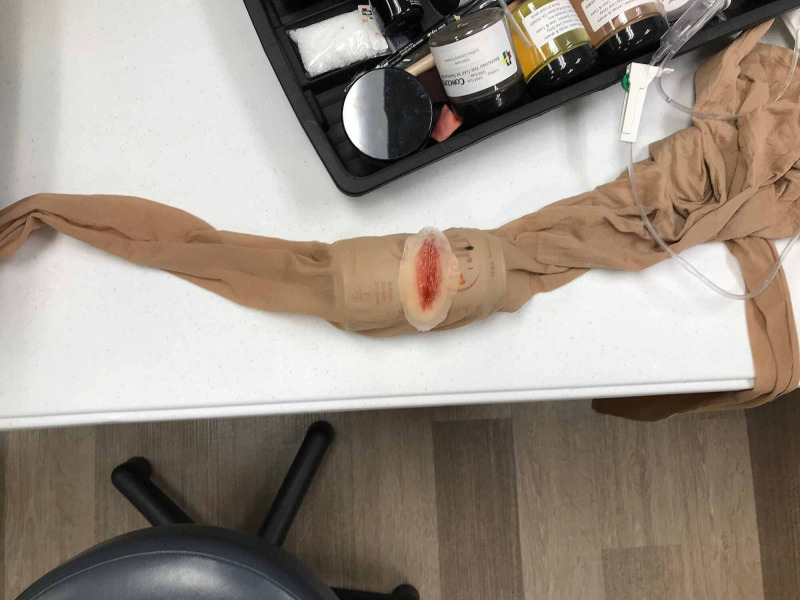
Bleeding wound set-up The image shows the use of a soda can and nylon stockings to fabricate a bleeding laceration that can be applied to a SP. A soda can is inserted into the nylon stockings as a placeholder as adhesive binds a silicone laceration injury to the stockings. Intravenous tubing ran through the inside of the nylon material and is connected to a bag of prop blood and fashioned to the silicone piece. The soda can then be removed, and the nylon apparatus can be applied to the extremity of a SP. SP, Standardized patient.

## Appendices

Appendix A: Script

Appendix B: Case flow

Appendix C: Labs

Appendix D: Fabrication of bleeding wound
